# AurkA controls self-renewal of breast cancer-initiating cells promoting wnt3a stabilization through suppression of miR-128

**DOI:** 10.1038/srep28436

**Published:** 2016-06-24

**Authors:** V. Eterno, A. Zambelli, L. Villani, A. Tuscano, S. Manera, A. Spitaleri, L. Pavesi, A. Amato

**Affiliations:** 1 Lab of Experimental Oncology & Pharmacogenomics IRCCS Fondazione “Salvatore Maugeri”, Pavia, Italy; 2 Unit of Oncology, IRCCS S. Maugeri Foundation, 27100 Pavia; 3 Unit of Pathology, IRCCS S. Maugeri Foundation, 27100 Pavia

## Abstract

AurkA overexpression was previously found in breast cancer and associated to its ability in controlling chromosome segregation during mitosis, however whether it may affect breast cancer cells, endorsed with stem properties (BCICs), is still unclear. Surprisingly, a strong correlation between AurkA expression and β-catenin localization in breast cancer tissues suggested a link between AurkA and Wnt signaling. In our study, AurkA knock-down reduced wnt3a mRNA and suppressed metastatic signature of MDA-MB-231 cells. As a consequence, the amount of BCICs and their migratory capability dramatically decreased. Conversely, wnt3a mRNA stabilization and increased CD44^+^/CD24^low/−^ subpopulation was found in AurkA-overexpressing MCF7 cells. *In vivo*, AurkA-overexpressing primary breast cancer cells showed higher tumorigenic properties. Interestingly, we found that AurkA suppressed the expression of miR-128, inhibitor of wnt3a mRNA stabilization. Namely, miR-128 suppression realized after AurkA binding to Snail. Remarkably, a strong correlation between AurkA and miR-128 expression in breast cancer tissues confirmed our findings. This study provides novel insights into an undisclosed role for the kinase AurkA in self-renewal and migration of BCICs affecting response to cancer therapies, metastatic spread and recurrence. In addition, it suggests a new therapeutic strategy taking advantage of miR-128 to suppress AurkA-Wnt3a signaling.

Despite the latest progresses, chemoresistance remains a critical hurdle which frequently leads to failure of anti-tumor therapies and relapse in breast cancer patients.

Both chemoresistance and tumor relapse seem to be dependent on a small population of cancer cells in the bulk of the tumor, endorsed with stem cells properties and named Cancer Initiating Cells (CICs)^1^. Recent studies have provided strong support for this hypothesis identifying CICs in several human cancers^2^,^3^,^4^,^5^,^6^.

Similarly to stem cells of mammary gland, from which they seem to originate, BCICs (CICs from the Breast) show a great plasticity and are able to self-renew^7^. They are identified by expression of some stem markers, as CD44 and CD24, and activation of the detoxifying enzyme, as ALDH. In particular, *in vivo* experiments suggest the idea that CD44^+^/CD24^low/−^ and ALDH^+^ BCICs retain a great tumorigenic potential making them able to form new tumors, even at very low concentration^2^,^8^. For this reason, it is commonly accepted that CICs, rather cancer cells of the bulk of the tumor, may account for tumor relapse.

In addition, it was suggested that conventional chemotherapies and radiotherapies are able to destroy the majority of cancer cells but are ineffective in targeting CICs, likely due to several resistance mechanisms (innate or acquired at later stage), making them refractory.

In light of the CICs theory, a further advance in cancer treatment would be provided by the identification of key molecules controlling the unique properties of CICs populations and, therefore, the development of CIC-related therapies which potentially would be suitable to treat different kind of tumors.

The Wnt pathway plays a fundamental role in proper mammary gland development, regulating self-renewal of stem-progenitors cells^9^. Moreover, nuclear accumulation of β-catenin is considered a trigger for transcription of genes implicated in self-renewal of CICs^10^,^11^.

However, contrasting evidences showed that overexpression of Wnt1, Wnt3a and Wnt7a promoted hyperplasia of mammary gland^12^, in contrast Wnt7b and Wnt5a failed to show a tumorigenic role in mice^13^, suggesting that each Wnt member may activate different signaling pathways depending on the cellular context.

Because of this complexity, the role of Wnt pathway in breast cancer and metastasis remains still uncleared.

Here, we show a post-transcriptional regulation of wnt3a by AurkA and reveal a novel role for the mitotic kinase in regulation of BCICs self-renewal and their metastatic properties.

For several years, AurkA has been known for a key role in centrosome duplication and chromosome segregation during mitosis^14^. Moreover, it is frequently amplified/mutated in several human cancers^15^,^16^,^17^,^18^,^19^,^20^ affecting their response to anti-tumor therapies and relapse^21^,^22^,^23^,^24^. Altogether these findings suggest that AurkA may contribute to tumor chemoresistance and metastatic spread^14^,^24^,^25^,^26^,^27^; however, whether it may have a role in controlling the amount of BCICs and their stem cells properties is still unclear.

Surprisingly, our findings suggest that AurkA regulates self-renewal, migratory activity and metastatic signature of BCICs through Wnt3a/β-catenin pathway. Indeed, we show that AurkA favors wnt3a mRNA stabilization in BCICs inhibiting miR-128.

Recently, miR-128 was found deleted in several human tumors. Previous data show that miR-128 may regulate wnt3a mRNA to promote differentiation of rat mesenchymal stem cells into neural cells^28^. In breast cancer, miR-128 inhibits expression of some stem gene as BMI1, CSF1, KLF4, LIN28A, NANOG^29^, however the molecular mechanisms still remain unknown.

Here we show that AurkA may suppress miR-128 expression through activation of Snail. Moreover, our study shows a strong correlation between AurkA and miR-128 expression in breast clinical isolates (N = 32) which further supports our findings.

Collectively our data reveal a new undisclosed role for the kinase AurkA in maintaining of BCICs. Moreover, our study suggests AurkA/miR-128/Wnt3a axys as a druggable target to inhibit chemoresistance and recurrence in breast cancer.

## Results

### AurkA overexpression is associated with β-catenin nuclear/cytoplasmic localization

AurkA overexpression/amplification was found in several human cancers. In breast cancer, some evidences sustain it is associated to basal-like phenotype^23^,^30^, others suggest it may be a marker for progression and outcome of luminal-like subtype^31^.

**Figure 1:**
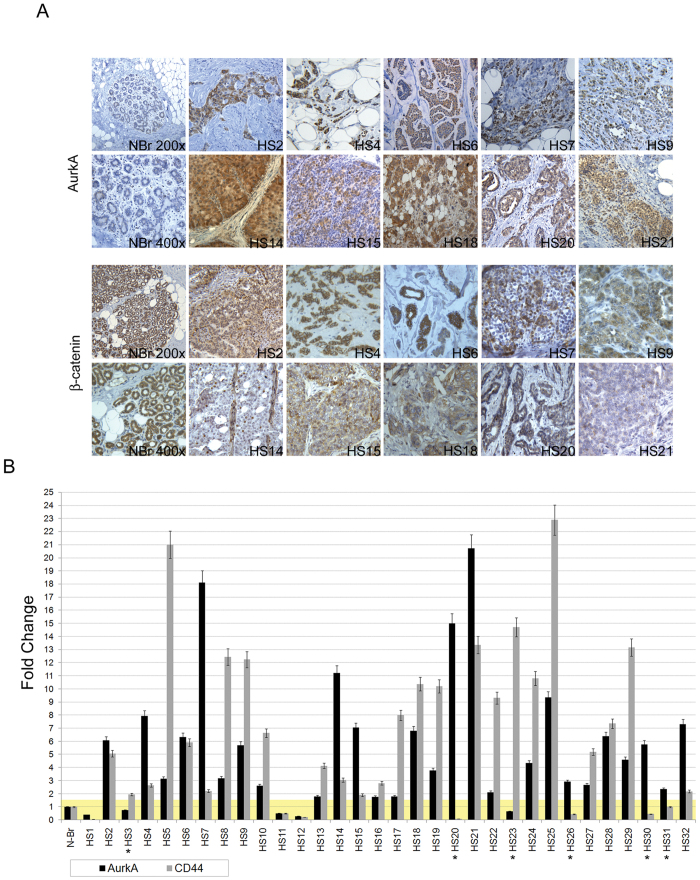
Aurka overexpression is associated with β-catenin nuclear/cytoplasmic localization. (**A**) Representative images of AurkA or β-catenin staining in 10 breast cancer tissues (HS2, HS4, HS6, HS7, HS9, HS14, HS15, HS18, HS20, HS21). Magnification 400X. Representative images of a normal breast tissue at 200X or 400X magnification were showed as controls (NBr 200X and NBr 400X). Nuclei were counterstained with Hematoxylin. B) Relative expression of AurkA (black bars) and CD44 (grey bars) in 32 breast cancer tissues (HS1-32). AurkA and CD44 fold changes result after normalization with normal breast tissue (N-Br). Data results from triplicate (±standard error) of each sample.

We analyzed the expression of AurkA kinase in 89 breast cancer patients by immunohistochemistry (IHC) (Fig. 1A). A significant positive staining of the kinase was found in 41 out 89 breast cancer patients (46.07%). However no correlation was found between AurkA overexpression and clinical and pathological features as grading (P_value_ = 0.759), Ki67 (P_value_ = 0.574), tumor size (P_value_ = 0.553) or linfonodal status (P_value_ = 0.107) in N = 89 breast cancer samples (Table 1).

Similar statistical analyses were performed to assess a correlation with breast cancer subtypes. On the basis of hormone receptors (ER and Her2), breast cancers were grouped in Luminal (ER-positive, 24/89), Her2+ (Her2-positive, 27/89) and TN (Triple Negative, 38/89). We found that 54.17% (13/24) among Luminals, 59.26% (16/27) among Her2+ and 31.58% (12/38) among TN, respectively, showed increased levels of AurkA in comparison with normal breast tissues (Table 1). However, no significant correlation was found between AurkA overexpression and any of the breast cancer subgroups (P_value_ = 0.0568) (Table 1).

Surprisingly, a strong correlation was found after evaluation of cellular localization of β-catenin (P_value_ = 0.00001) by IHC. Noteworthy, we found that breast cancer samples showing increased levels of AurkA, lost cortical β-catenin (Fig. 1A).

A β-catenin dislocation was previously associated to high aggressive breast cancer cells^32^. In addition, nuclear β-catenin suggests the activation of the Wnt signaling^33^.

Interestingly, we found that breast cancer samples, overexpressing AurkA (>1.5 fold-change in comparison with normal breast), similarly showed increased levels of CD44 as revealed by Q-PCR. Conversely, low levels of AurkA mRNA (<1.5 fold-change) corresponded to low levels of CD44. This significant correlation (P_value_ = 0.00001) was found in 26 out 32 breast cancer samples (Fig. 1B).

### AurkA controls BCICs through regulation of Wnt3a/β-catenin

The discovery that overexpression of AurkA correlated with nuclear/cytoplasmic localization of β-catenin and increased expression of CD44 suggested that AurkA may have a role in controlling BCICs.

To verify this hypothesis, we modulated AurkA expression in breast cancer cell lines and evaluated the effects on BCICs.

**Figure 2:**
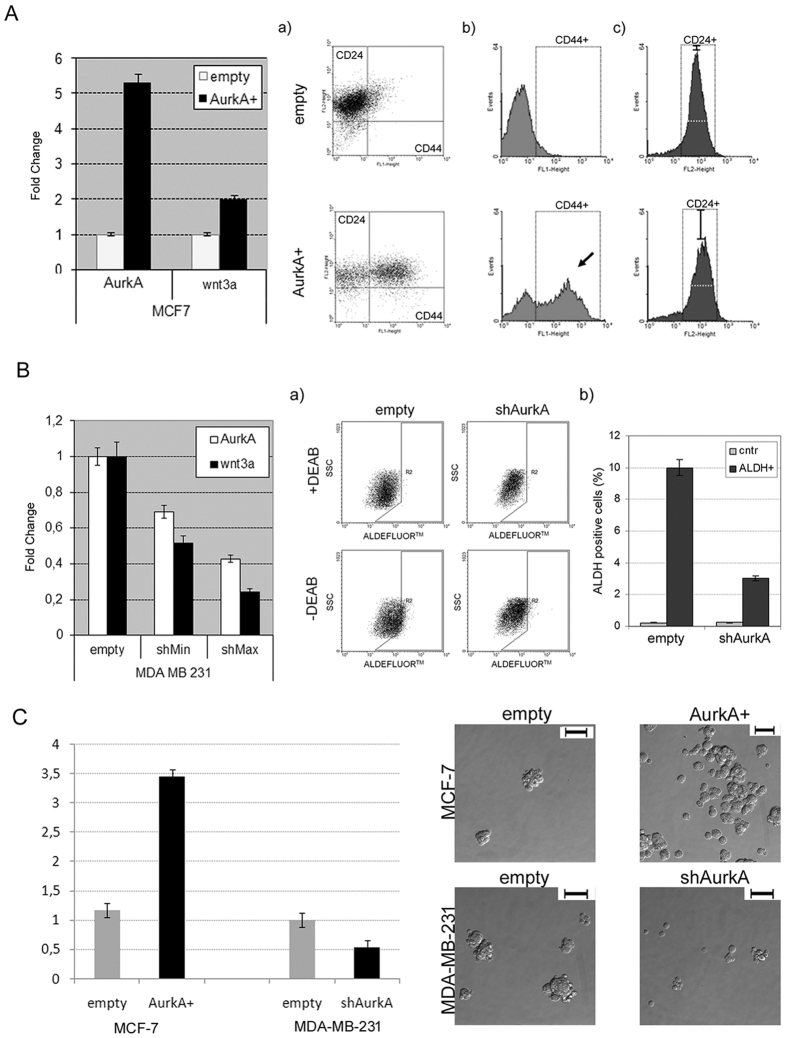
Aurka controls breast cancer stem cells through regulation of Wnt/β-catenin. (**A**) On the left, relative quantification of AurkA and wnt3a in MCF-AurkA+ cells (AurkA). MCF-7 carrying empty vector (empty) were considered as control. On the right, (a) Cytofluorimetric panels show distribution of MCF-AurkA or MCF-empty cells depending on CD44-CD24 staining; (b,c) Distribution of CD44+ or CD24+ subpopulation, respectively, in MCF-empty (top panels) and MCF-AurkA cells (bottom panels). Black arrow in (b) highlights the increase of CD44+ cells in AurkA overexpressing cells (AurkA+). Black bar in (c) highlights the decrease of CD24 expression cells in AurkA overexpressing. (**B**) On the left, relative quantification of AurkA and wnt3a after AurkA silencing at low (sh8, shMin) or high (sh5, shMax) efficiency in MDA-MB-231 cells. MDA-MB-231 cells carrying empty vector (empty) were considered as control. (a) On the right, cytofluorimetric panels showing distribution of MDA-shAurkA versus MDA-empty cells depending on ALDH-expression (bottom panels). +DEAB empty or shAurkA were negative controls (top panel). (b) ALDH positive cells were scored as reported in the right graph. (**C**) MFE (Mammosphere Forming Efficiency) valued in AurkA+ MCF-7 or in shAurkA MDA-MB-231. Control cells are indicated as empty. Representative images for each sample were showed at 200X magnification (Scale bar: 800 μm). Data are representative of biological triplicates.

Ectopic expression of AurkA was induced in MCF7 cells (hereinafter MCF-AurkA+, for brevity) (Fig. 2A). Conversely, inhibition of AurkA expression was carried out in MDA-MB-231 (hereinafter MDA-shAurkA, for brevity) after transfection with two different shRNA, resulting in 30% (shMin) or 60% decrease of AurkA expression (shMax) (Fig. 2B).

Surprisingly, we found that modulating AurkA expression we were able to change expression levels of wnt3a (Fig. 2A,B). Accordingly, we found an increase in the amount of CD44^+^/CD24^low/−^ subpopulation in MCF-AurkA+, (Fig. 2A,a), due to a marked increase in CD44-positive cells (Fig. 2A,b) and a decrease of CD24-positive cells (Fig. 2A,c). Moreover, sphere-forming assay revealed that MCF-AurkA+, increased their ability to grow as mammospheres in comparison with control cells (Fig. 2C).

As well, AldeFluor assay showed a significant decrease of ALDH-positive cells in MDA-shAurkA cells suggesting that inhibition of AurkA affected stem-like subpopulation (Fig. 2B, a and b), as confirmed by a marked decrease in mammospheres formation when MDA-shAurkA cells were grown in low-adhesion conditions (Fig. 2C).

**Figure 3:**
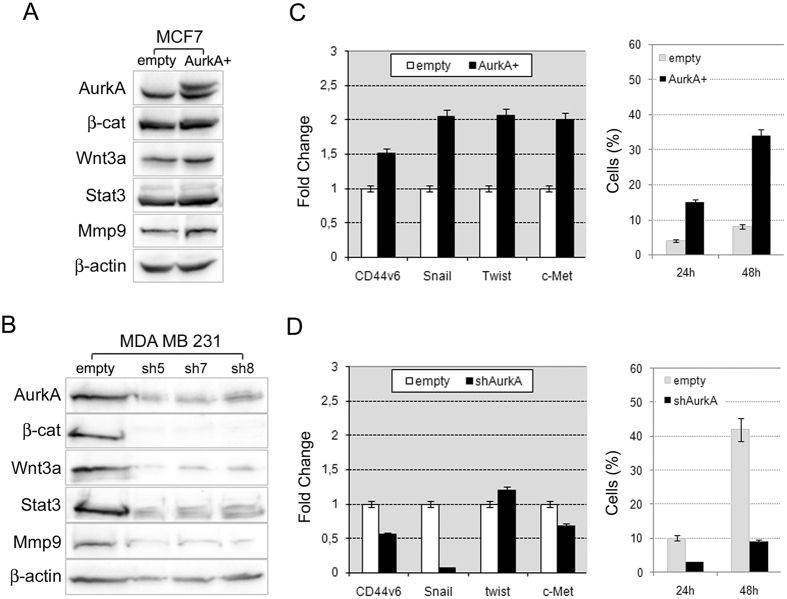
Aurka controls breast cancer stem cells through regulation of Wnt/β-catenin. (**A**) AurkA overexpression in MCF-7 cells promoted AurkA protein stabilization and increased levels of β–catenin, Wnt3a, Stat3 and Mmp9 proteins; (**B**) conversely, AurkA knock-down dramatically reduced AurkA protein levels as well as levels of β–catenin, Wnt3a, Stat3 and Mmp9 proteins. β–actin is a loading control. (**C**) AurkA overexpression associated with a metastatic signature in MCF-AurkA cells as suggested by increased expression of CD44v6, Snail, Twist and c-Met and increased migratory activity (right graph) in comparison with control cells (empty). (**D**) In contrast, MDA-shAurkA cells lost that signature showing a repression of CD44v6, Snail and c-Met and a marked inhibition of migratory activity (right graph) in comparison with control cells (empty).

The involvement of AurkA in regulation of BCICs was further confirmed by western blot analysis showing increased wnt3a and β-catenin in MCF-AurkA cells (Fig. 3A). Conversely β-catenin degradation and undetectable wnt3a levels were found in MDA-shAurkA (Fig. 3B). Those data strongly suggested that the kinase may control BCICs through the Wnt3a/β-catenin axis.

In addition, western blot analyses revealed a link between AurkA and some proteins involved in cellular migration and metastasis as Mmp9 and Stat3. In particular, we found that AurkA overexpression promoted stabilization of Mmp9 and Stat3 (Fig. 3A), conversely AurkA silencing severely affected the amount of the same proteins (Fig. 3B).

Collectively, these data suggest that AurkA may take part in mechanisms underpinning self-renewal and metastatic properties of BCICs.

Actually, this hypothesis was further supported by Q-PCR revealing increased expression of EMT and migratory genes such as CD44v6, Snail, Twist and c-Met in MCF-AurkA+ cells (Fig. 3C), whereas a significant decrease (except for twist) in MDA-shAurkA cells (Fig. 3D). Moreover, AurkA overexpression promoted migration of MCF7 cells which usually exhibit a low aptitude to migrate in invasion assays (Fig. 3C). Conversely, the marked migratory activity of MDA-MB-231 cells was inhibited after AurkA knock-down (Fig. 3D).

### Tumorigenic primary breast cancer cells show higher levels of AurkA

To assess if AurkA may promote a more aggressive phenotype *in vivo*, we isolated primary breast cancer cells (named KBr1, KBr2, KBr3 and KBr4) from 4 different mastectomized breast cancer patients and injected them into immune-compromised mice.

**Figure 4:**
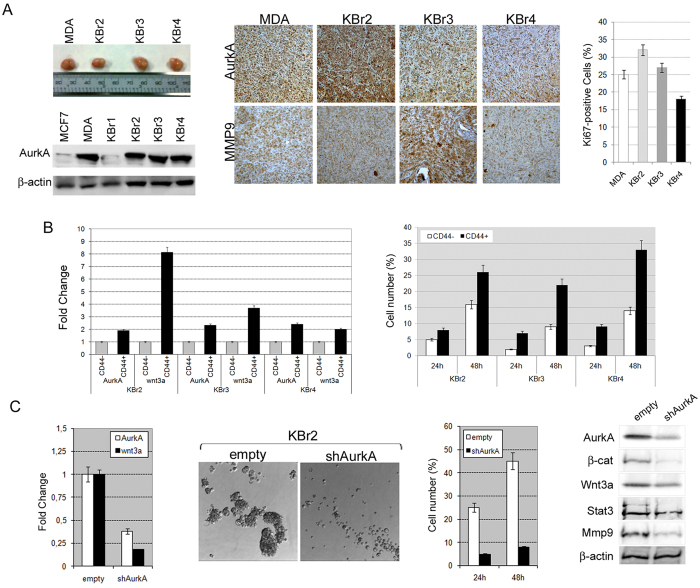
Tumorigenic primary breast cancer cells showed higher levels of AurkA. (**A**) Primary breast cancer cells showing increased expression of AurkA (KBr2, KBr3, KBr4, bottom panel) exhibited a more aggressive phenotype evaluated as the capacity to generate tumors after injection into immune-compromised mice (top panel), expression of Mmp9, a metastatic marker, and Ki67 high levels (>10%). (**B**) CD44-positive cells from KBr2, KBr3 and KBr4 showed higher AurkA and wnt3a expression levels in comparison with CD44-negative cells (control), and increased migratory activity evaluated at 24 and 48 hours from seeding (respectively 24 h and 48 h). (**C**) AurkA knock-down (shAurkA) severely affected AurkA and wnt3a cDNAs levels, adhesion-indipendent growth and migratory activity in KBr2 cells as compared with control cells (empty). Moreover, it reduced levels of AurkA, β–catenin, Wnt3a, Stat3 and Mmp9 proteins.

We analyzed correlation between AurkA expression and tumorigenic properties of cancer cells. We found that only 3 samples (KBr2, KBr3 and KBr4), showing a high expression of the kinase (similar to MDA-MB-231 cells), were tumorigenic. In contrast, AurkA-low expressing KBr1 failed to generate tumors after injection *in vivo*, as MCF7 cells (Fig. 4A).

Moreover, analyses on xenografts confirmed a correlation between AurkA overexpression and a more aggressive behaviour *in vivo* as revelead by positive MMP9 staining and high Ki67 values (Fig. 4A).

Actually, we found that the CD44-positive subpopulation in primary breast cancer cells (KBr2, KBr3 and KBr4), showed higher levels of AurkA and wnt3a mRNAs as well as increased migratory ability, compared to CD44-negative cells (Fig. 4B).

Expression of wnt3a, adhesion-independent growth and migration were severely impaired after inhibition of AurkA in KBr2 cells (Fig. 4C, shAurkA), suggesting inhibition of the Wnt3a/β-catenin signalling in KBr2-shAurkA as supported by reduced wnt3a, β-catenin, Stat3 and Mmp9 proteins (Fig. 4C).

### AurkA promotes stabilization of wnt3a mRNA through repression of miR-128

Our findings suggest a new role for AurkA, which proves to be able to control BCICs and, likely, aggressiveness of breast cancer. Next we wanted to unravel the molecular mechanisms underpinning this new role of the kinase.

Some evidences showed that AurkA may control the expression of some genes through regulation of several microRNAs (miRNAs), namely miR-21 in hepatocellular carcinoma^34^. In breast cancer, it was found that AurkA control the miR17-92 cluster through regulation of E2F1 transcription factor^35^.

Here, we hypothesized that AurkA may control the expression of a miRNA which should act as a repressor for wnt3a. An in-depth analysis by TargetScan 7.0 Software allowed to identify three different miRNAs as specific regulators for wnt3a, unknown to be AurkA target: miR-15, miR-16 and miR-128.

**Figure 5:**
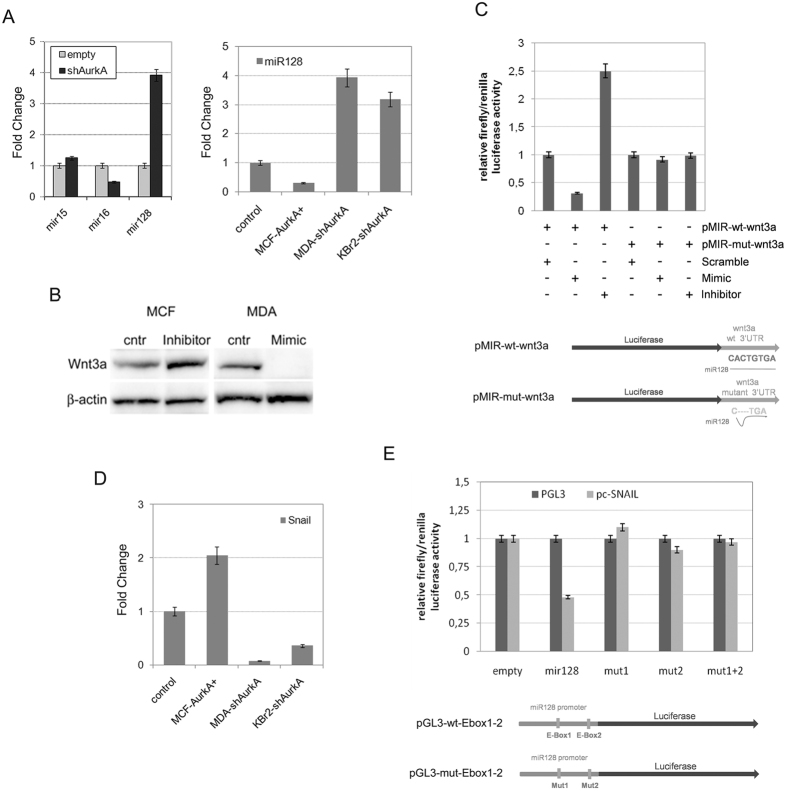
Aurka promote stabilization of wnt3a through repression of miR-128. (**A**) Q-PCR showed that AurkA knock-down in MDA-MB-231 cells promoted an increase of mir128 expression while reduced levels of mir15 and mir16. Effectively, there was a mechanism of miR128 inhibition by AurkA as revealed by Q-PCR after AurkA overexpression in MCF-7 (MCF-AurkA+) cells of knock-down in MDA-MB-231 and KBr2 cells (MDA-shAurkA and KBr2-shAurka). (**B**) miR-128 controls wnt3a. miR-128 inhibitors promoted Wnt3a protein stabilization in MCF-7 (lane 2), conversely specific miR-128 mimics (lane 4) repressed Wnt3a in MDA-MB-231 cells, untreated MCF-7 or MDA-MB-231 cells were considered as control (lane 1 and 3, respectively). (**C**) miR128 bind to wnt3a 3′UTR. Luciferase activity increased when HEK-293T cells were co-transfected with pMIR-wt-wnt3a and miR128 inhibitors (3^rd^ bar), decreased in presence of pMIR-wt-wnt3a and mimics (mimicking miR128 activity, 2^nd^ bar). In contrast, it was not affected in control cells co-transfected with pMIR-wt-wnt3a and miR128 scramble (1^st^ bar) or co-transfected with pMIR-mut-wnt3a (carrying mutated 3′UTR of wnt3a) and miR128 scramble, mimics or inhibitor (respectively 4^th^, 5^th^, 6^th^ bars). At the bottom a schematic view of pMIR-wt-wnt3a and pMIR-mut-wnt3a. Data are representative of biological triplicates. (**D**) AurkA expression affected Snail transcription. Snail cDNA increased after AurkA overexpression in MCF-7 (MCF-AurkA+) cells and increased after AurkA knock-down in MDA-MB-231 and KBr2 cells (MDA-shAurkA and KBr2-shAurka). (**E**) Increased luciferase activity was found when 293T cells were co-transfected with a vector carrying snail and pGL3-wt-Ebox1–2 (2^nd^ gray bar). Conversely, luciferase activity was unaffected when 293T were co-transfected with a snail-vector and pGL3-mut-Ebox1–2 (carrying mutated Ebox1, Ebox2 or both, respectively 3th, 4^th^ and 5^th^ gray bars). Control cells, co-transfected with pGL3-wt-Ebox1–2 or pGL3-mut-Ebox1–2 with pGL3 (missing Snail gene) showed basal luciferase expression.

By Q-PCR analysis, we were able to assess a correlation between miR-128 and AurkA. Basically, we found that AurkA knock-down in MDA-MB-231 was ineffective for stabilization of miR-15 and reduced miR-16 expression, whereas, induced a significant increase in miR-128 levels (Fig. 5A, left graph).

Accordingly to our previous hypothesis, if AurkA controls transcription of wnt3a through a repressive activity of a miRNA, a marked increased expression of this miRNA should be found after AurkA knock-down. As a consequence, we considered miR128 as a target of AurkA, excluding miR15 and miR16.

Indeed, we confirmed a marked decrease of miR-128 in MCF-AurkA cells and increased expression in both MDA-shAurkA and KBr2-shAurkA (Fig. 5A, right graph). Moreover, we showed that Wnt3a protein levels increased after suppression of endogenous miR-128 expression in MCF7 cells (Fig. 5B, inhibitor vs cntr). Conversely, we repressed Wnt3a stabilization in MDA-MB-231 cells after stimulating miR-128 activity with specific mimics (molecules simulating miR-128 activity, Fig. 5B, Mimic vs cntr).

A miR-128 specific binding to wnt3a mRNA was proved by luciferase assay. A vector, carrying the 3′UTR region of wnt3a downstream luciferase gene, was transfected in HEK-293T cells (Fig. 5C, pMIR-wt-wnt3a). We observed a decreased luciferase activity in presence of mimics, whereas luciferase activity was restored by an inhibitor specific for endogenous miR-128 (Fig. 5C). Conversely, neither mimics nor inhibitor were able to affect luciferase activity when HEK-293T cells were transfected with a vector carrying a mutated 3′UTR region (Fig. 5C, pMIR-mut-wnt3a).

### Snail mediates repression of miR-128 in response to AurkA overexpression

Given our findings proving that miR-128 is able to repress wnt3a at post-transcriptional level, we hypothesized whether a transcription factor may exist which is able to repress miR-128 activity on a fashion that is dependent on AurkA.

Previous findings suggested Snail as a repressor for miR-128^29^. Based upon this first evidence, we evaluated if snail may be a more likely candidate.

Q-PCR confirmed a correlation between AurkA and Snail, showing increased snail mRNA levels in MCF-AurkA cells and, conversely, a marked decrease in both MDA-shAurkA and KBr2-shAurkA cells (Fig. 5D).

A luciferase assay was carried out to verify a site-specific binding of Snail to E-Box1 and E-Box2 of miR-128 promoter (Fig. 5E, pGL3-miR-128), as previously described in ref. 29.

Decreased luciferase activity was found when HEK-293T cells were co-transfected with a vector carrying snail-cDNA and pGL3-wt-Ebox1–2 (Fig. 5E, pGL3-wt-Ebox1–2). Conversely, luciferase activity was unaffected when co-transfection was performed substituting pGL3-wt-Ebox1–2 vector with one carrying mutated Ebox1 (gray bar, mut1) or Ebox2 (gray bar, mut2) or both (gray bar, mut1 + 2) (pGL3-mut-Ebox1–2, Fig. 5E).

Our data confirm that miR-128 is a target of Snail in our experimental model, in addition they suggest that the transcription factor may be regulated by the kinase AurkA.

**Figure 6:**
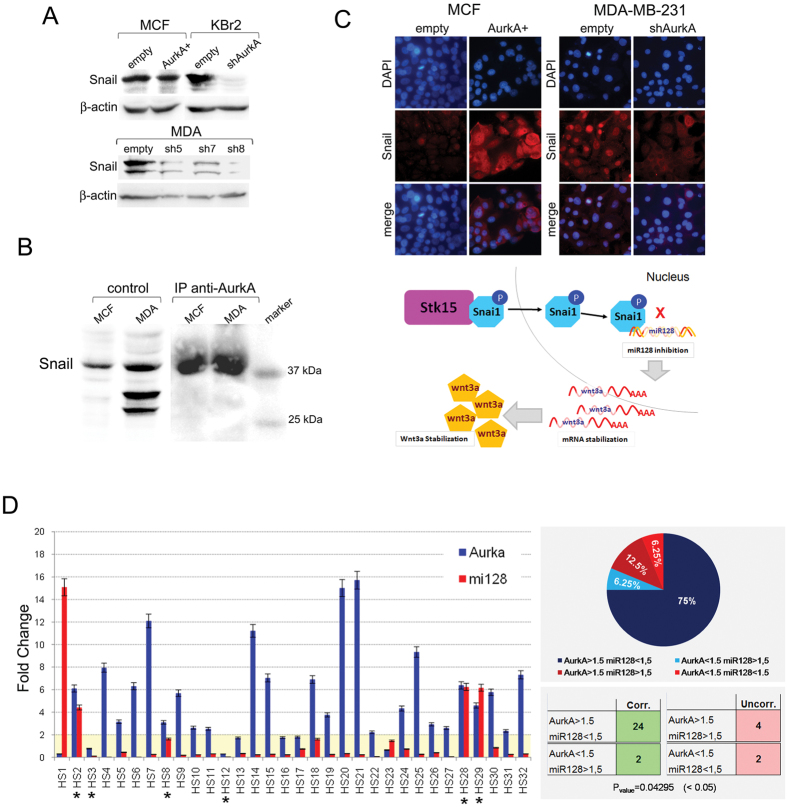
Snail mediates repression of miR-128 in response to Aurka overexpression. (**A**) AurkA overexpression does not promote Snail protein increase as found in MCF-AurkA (AurkA+) versus control cells (empty) Evaluation of Snail protein levels in MCF-7 and MCF-AurkA. In contrast, AurkA knock-down severely impaired Snail protein levels in MDA-shAurkA (sh5, sh7, sh8) and KBr2-shAurkA (shAurkA) in comparison with control cells (empty). (**B**) AurkA antibody pulls down Snail in MCF7 and MDA-MB-231 cells after a co-immunoprecipitation assay (IP anti-AurkA). As control, protein lysates from both cell lines were tested for Snail protein levels (control). A marker was loaded in the last lane as a control of molecular weight. (**C**) AurkA overexpression in MCF-7 cells induced nuclear translocation of Snail (MCF, AurkA+). Control cells show a moderate cytoplasmic staining (MCF, empty). Nuclear accumulation of Snail in MDA-MB-231 cells (MDA, empty) was inhibited by AurkA knock-down (MDA, shAurkA). Nuclei were counterstained with DAPI. Collectively our findings supports a signaling pathway showing that AurkA may promote nuclear translocation of Snail to repress miR-128 gene transcription. As results, wnt3a mRNA accumulates increasing Wnt3a protein levels. (**D**) On the left, Q-PCR confirmed association between AurkA and miR-128 levels in 32 human tissues from breast cancer patients. Asterisk highlights samples were association is missing. On the right, Graphic representation of of samples (percentage, %) showing a strong correlation AurkA-mir-128 (AurkA > 1.5/miR-128 < 1.5 and AurkA < 1.5/miR-128 > 1.5), or where AurkA-miR-128 correlation is missing (AurkA > 1.5/miR-128 > 1.5 and AurkA < 1.5/miR-128 < 1.5).

Indeed, we found that MDA-shAurkA and KBr2-shAurkA showed decreased snail in comparison with control cells (Fig. 6A), surprisingly no significant change was found in MCF-AurkA+ cells which showed Snail protein levels similar to control cells (Fig. 6A).

This finding suggest that AurkA likely controls Snail through regulation of its nuclear localization. A co-immunoprecipitation assay confirmed that Snail is a direct target of the kinase in MCF-7 and MDA-MB-231 cells (Fig. 6B).

Moreover, immunofluorescence analyses suggested that AurkA-Snail Interaction may affects subcellular localization of Snail. Indeed, we found that AurkA overexpression in MCF-7 cells induced nuclear translocation of Snail, in comparison with control cells showing a moderate staining for Snail in the cytoplasm (Fig. 6C left panel).

In contrast, AurkA knock-down in MDA-MB-231 cells induced a cytoplasmic accumulation of Snail in comparison with control cells, showing a nuclear localization of the protein (Fig. 6C, right panel).

Those data strongly suggest that AurkA may phosphorylates Snail promoting nuclear translocation of the transcription factor. As a consequence, Snail inhibits transcription of miR-128 resulting in Wnt3a mRNA stabilization and protein accumulation (Fig. 6C, bottom panel).

Consistent with this new role for AurkA in controlling the stem-like subpopulation through Wnt3a/β-catenin signaling, a direct consequence seems that AurkA may affect chemoresistance and recurrence in cancer patients.

This hypothesis was further confirmed by a strong correlation (P_value_ = 0.04295) between AurkA and miR-128 expression in a subset of primary ductal invasive breast cancers (N = 32) by IHC. Breast tumors showing higher expression of AurkA (fold change >1.5), expressed miR-128 at very low levels (fold change <1.5), conversely tumors showing low levels of AurkA (fold change <1.5), exhibited higher levels of miR-128 (fold change >1.5) (Fig. 6D). This correlated expression was found in 26 out 32 breast cancers (75% + 6.25% = 81.25%) supporting the hypothesis of AurkA-miR-128-Wnt3a/β-catenin signaling also in clinical specimens (Fig. 6D).

## Discussion

In the last decade, growing evidences have strongly supported that a subset of cells within the tumor bulk, referred as Cancer Initiating Cells (CICs) may account for tumor growth, resistance to anti-cancer treatments and metastatic spreads. Hence, the amount of CICs within the tumor bulk dramatically affects survival and outcome of cancer patients.

So far, many studies have been addressed to the identification of “druggable” pathways with key roles in self-renewal and chemoresistance of CICs.

Our study shows a novel role for AurkA in maintaining Breast Cancer Initiating Cells (BCICs) through a pathway involving a Snail-miR-128-wnt3a/β-catenin axis as signaling mechanism (Fig. 6C, bottom picture).

So far oncogenic properties of AurkA has been attributed to its pivotal role during mitosis, our data suggest that AurkA control self-renewal (Fig. 2), metastatic signature and migratory activity of BCICs (Fig. 3).

In this study, we induced ectopic expression of AurkA in MCF-7 cells, a low-aggressive breast cancer cell line with low metastatic potential. We found that AurkA overexpression increased the BCICs subpopulation, identified as CD44^+^/CD24^low/−^ cells (Fig. 2A), able to grow as mammospheres (Fig. 2C); moreover it was associated with increased wnt3a mRNA and protein levels (Figs 2A and 3A), and a metastatic signature as expression of Twist, Snail, c-Met, CD44v6, MMP-9 and Stat3 (Fig. 3A,C).

Those effects were severely impaired after AurkA knock-down in MDA-MB-231 cells, a very aggressive and metastatic breast cancer cell line. MDA-shAurkA cells lost their metastatic signature and migratory activity (Fig. 3B,D). The amount of BCICs, identified as ALDH-positive cells (Fig. 2B, a and b), dramatically decreased and, surprisingly, they showed low levels of wnt3a mRNA and protein (Figs 2B and 3B).

Altogether those findings strongly suggested that AurkA may control self-renewal and invasive capacity of BCICs, contributing to a worse outcome in breast cancer patients.

This role seems to involve the activation of canonical Wnt3a pathway as suggested by the association between AurkA overexpression and nuclear/cytoplasmic localization of β-catenin (P_value_ = 0.00001) found in 89 breast cancer primary tumors (Fig. 1A) as well as the association between AurkA expression and β-catenin stabilization found in our experimental model (western blot in Fig. 3A,B).

Nuclear β-catenin, previously associated to high aggressive breast cancer cells^32^, its considered a marker for Wnt signaling activation and self-renewal of cancer cells. This hypothesis was further corroborated by the correlation between AurkA and CD44, a marker for breast cancer stem cells (P_value_ = 0.00001) in 26 out 32 breast cancer samples (Fig. 1B).

Those evidences were confirmed also in primary breast cancer. Indeed, we found that tumorigenic KBr2, KBr3 and KBr4 showed higher levels of AurkA, similar to MDA-MB-231 cells. In addition a positive staining for MMP9 and increased Ki67 values suggested that they may have a more aggressive phenotype (Fig. 4A). In contrast, KBr1, displaying AurkA levels similar to MCF-7 cells, failed to be tumorigenic after injection (Fig. 4A).

Moreover, analysis of the CD44-positive cells in KBr2 cells, showed higher levels of Wnt3a and AurkA (Fig. 4B) as well as marked migratory activity and adhesion-independent growth, which were severely impaired after AurkA knock-down as shown in Fig. 4C.

Those findings further confirm the role of AurkA in sustaining self-renewal and aggressive behavior of BCICs through activation of Wnt3a/β-catenin signalling.

Actually, we show a post-transcriptional control of wnt3a by AurkA. Indeed, the kinase overexpression promoted a dramatic decrease of miR-128.

It is well known that miRNAs may influence carcinogenesis acting as oncogenes or tumor suppressors, although the molecular mechanisms are still unclear.

Here we found that miR-128 is an inhibitor of self-renewal and metastatic signature of BCICs, because it inhibits stabilization of wnt3a mRNA, in a fashion that is dependent on AurkA kinase.

However AurkA is not a transcription factor, so it is conceivable that the kinase may regulate miR-128 gene expression controlling a transcription factor. Previous findings suggested Snail as a repressor for miR-128^29^.

Indeed, a luciferase assay confirmed a repressive activity of Snail on miR-128 gene in our system, in addition showed that Snail activity changed accordingly to Aurka overexpression/knock-down (Fig. 6A).

Snail appears to be a direct target of AurkA Indeed, here we show for the first time that AurkA binds to Snail, as revealed by co-immunoprecipitation assay (Fig. 6B). Moreover, immunofluorescence analyses suggested that the kinase may promote nuclear translocation of snail and, as a consequence, its repressive activity on gene expression (Fig. 6C).

To be thorough, we notice that AurkA knock-down in MDA-MB-231 or in KBr2 cells reduced snail mRNA and protein levels (Figs 5C and 6A), however AurkA overexpression in MCF-7 cells was not followed by Snail protein stabilization (Fig. 6A), although they showed increased mRNA levels (Fig. 5D). Therefore, we consider that the effects we have found in Snail stabilization were a side-effect depending on a regulation loop between Snail and miR-128, as it was previously reported^29^.

In light of this study, we suggest that the oncogenic role of AurkA in breast cancers is not only depending by its key role during mitosis, but may include its capacity to control the “hard-core” of the tumor, that is the BCICs in the inner mass which account for response to cancer therapies, metastatic spread and recurrence.

This observation explains the association between AurkA overexpression and the worse clinical outcome previously reported for several human tumors as for breast cancer^21^,^23^,^31^.

In conclusion, we want to point out that the pivotal role of Aurka affecting BCICs self-renewal and invasive properties is depending on a Snail-miR-128-Wnt3a/β-catenin axys (Fig. 6C bottom picture).

This molecular mechanism is strongly supported also by the strong correlation between AurkA expression and miR-128 levels in 81,25% (26 out 32) (P = 0.04295) of breast cancer patients (Fig. 6D).

Finally, we suggest that targeting this signaling pathway would be helpful as a therapeutic strategy to treat chemo-resistance and metastatic spread in breast cancer patients.

## Materials and Methods

### Ethics Statement

Investigation has been conducted in accordance with the ethical standards and approved national and international guidelines. The study involving human tissues has been approved by Central Ethic Committee (CEC) at IRCCS Fondazione “Salvatore Maugeri”, Pavia. Clinical tumor specimens from post-surgery breast cancer patients were collected by the Institutional Biobank “Bruno Boerci”, after that written informed consent was obtained from each patient.

*In vivo* study carried out with mouse models was performed according to the approved international guidelines, in addition the experimental procedures involving animals were approved by Italian Ministry of Health.

### Clinical isolates

Formalin-fixed, paraffin-embedded (FFPE) tumor tissues from a cohort of 89 primary invasive ductal breast carcinomas were obtained. Histological typing, grading, staging and evaluation of estrogen receptor (ER) and progesterone receptor (PgR) status were performed as part of routine diagnostic protocol according to standard histopathologic techniques by the Unit of Pathology at IRCCS.

### Cell culture

Culture of breast cancer cell lines, isolation/culture of primary cells, invasion assay, MACS sorting were performed as previously described in ref. [30].

### Sphere-forming Assay

Breast cancer cell lines (MCF-AurkA, MDA-shAurkA or control cells, respectively, MCF-7 empty and MDA-MB-231 empty) were suspended each at a density of 500 cells/cm^2^ in 6-well ultra–low adherent plates (Corning) in DMEM/F12 supplemented with B-27® Serum-Free Supplement (Life technologies), bFGF (10 ng/ml, SIGMA) and EGF (20 ng/ml, SIGMA).

Sphere forming was monitored for 1 week. Mammospheres showing greater than 40 μm were scored, using an inverted light microscope DM5000B (Leica) equipped with a CCD camera and LAS software (Leica) for picture capture. Experiments were performed as triplicate and MFE (Mammosphere Forming Efficiency) was calculated as reported in^36^. Standard Deviation was calculated for each sample. Representative images were showed at 200X magnification.

### Immunohistochemistry

For immunohistochemistry on human breast cancer tissues and tumor xenografts, FFPE tumor specimens were processed to obtain 5 μm-thick slides. Epitope retrieval was performed in Citrate Buffer pH6 (DAKO) in a pre-warmed bath at 98 °C for 45 minutes. Primary antibody incubation was carried out at 4 °C ON. Primary mouse antibodies: AurkA (1:100, sc-25425, Santa Cruz Biotechnology) β-catenin (1:50, Sc-7963 Santa Cruz Biotechnology), MMP9 (1:100, Sc-21733 Santa Cruz Biotechnology). Normal breast tissues were used as control of staining.

Samples were analyzed on Leica DM1000 Microscope (Leica) equipped with LAS Software for image capture and analysis. For each slide a least 50 fields were analyzed.

For Ki67 counting, at least ten randomly selected regions for slides were analyzed and a minimum of 500 nuclei was counted for each sample. Representative images at 200X magnification has been shown.

### Immunofluorescence

Cells were seeded in coverslips (20 mm diameter) and allowed to reach 80% confluence, then fixed for 5 minutes with 100% ice-cold Methanol. After 10 minutes permeabilization with 0.1% Triton-X100 (Sigma-Aldrich), blocking buffer (0.1% BSA, Sigma-Aldrich) was added and slides incubated at RT for 1 h. Incubation with Anti-snail antibody (1:100, PA5–11923 Pierce) was allowed ON at 4 °C, followed by 1 h incubation at RT with anti-rabbit-Cy5 antibody (1:400, Jackson).

Samples were analyzed on Leica DM1000 Microscope (Leica) equipped with LAS Software for image capture and analysis. For each slide a least 50 fields were analyzed. Representative images at 200X magnification has been shown.

### Western blot analysis

SDS-PAGE was performed as previously described in^37^. Primary antibodies were: mouse monoclonal β-actin (1:400, Sc-47778 Santa Cruz Biotechnology), β-catenin (1:100, Sc-7963 Santa Cruz Biotechnology), MMP9 (1:200, Sc-21733 Santa Cruz Biotechnology), Wnt3a (1:200, sc-136163 Santa Cruz Biotechnology), rabbit polyclonal AurkA (1:400, sc-25425, Santa Cruz Biotechnology), Snail (1:100, PA5-11923 Pierce), Stat3 (1:200, sc-482 Santa Cruz Biotechnology). A representative picture of three independent experiments was reported.

### Q- PCR

Total RNA isolation, preparation of cDNA, Q-PCR and data analyses were performed as previously described in ref. 37. Probes for all genes (AurkA, wnt3a, miR-128, twist, snail, c-Met, CD44, CD44v6 and GAPDH and U6 small nuclear RNA as controls) areTaqMan® Gene Expression Assay (Thermo Fisher Scientific).

### *In vivo* experiments

*In vivo* experimentation carried out with mouse models were realized as previously described in ref. 37.

### Plasmid constructs and Luciferase assay

To prove that miR-128 specifically binds to wnt3a mRNA, a luciferase assay was carried out using pMIR-wt-wnt3a, a vector containing 3′UTR wild-type region of wnt3a downstream the firefly luciferase gene (Origene). As a control, a pMIR-mut-wnt3a vector containing mutated miR128-seed region of wnt3a 3′UTR was used. Mutagenesis in pMIR-wt-wnt3a was carried out using The QuikChange II XL site-directed mutagenesis kit (Stratagene), resulting in pMIR-mut-wnt3a.

HEK-293T cells were seeded in 48-well plates and transfected with 50 nM of microRNA-scramble or microRNA-128 mimics (both from Sigma-Aldrich) or microRNA-128 inhibitor (Thermo Fisher Scientific) and Renilla vector (25 ng, pRL-TK, Promega as internal control) together with 200 ng pMIR-wt-Wnt3a or pMIR-mut-Wnt3a. X-tremeGENE Transfection Reagent (Roche Applied Science) was used for transfection.

For miRNA promoter assay, a 1200 bp DNA fragment containing the E-box1and E-box2 upstream miR-128-2 precursor was amplified and cloned into the pGL3 Basic Luciferase vector (Promega) resulting in pGL3-wt-Ebox1–2. In-Fusion® HD Cloning Kit (Clontech) was used. To delete E-Box sequences, site-directed mutagenesis were performed by using the QuickChange II XL site-directed mutagenesis kit (Stratagene), resulting in pGL3-mut-Ebox1–2.

For miRNA promoter luciferase assay, HEK-293T cells were seeded and co-transfected as describe above using 100 ng pCMV6XL5 containing Snail full lenth cDNA and 100 ng pGL3-wt-Ebox1–2 or pGL3-mut-Ebox1–2 and 25 ng of Renilla vector (pRL-TK, promega).

Cells were harvested after 48 h from transfection and luciferase activity was measured using the Dual-luciferase Reporter assay (Promega) according to the manufacturer’s protocol.

### Production of lentiviral particles and infection

AurKA cDNA was cloned into pCDH-CMV-MCSEF1-copGFP lentiviral vector (System Biosciences), containing GFP (Green Fluorescence Protein) as reporter gene.

AurKA silencing was performed in MDA-MB-231 cells and in KBr2 primary culture with PLK01 lentiviral vector containing sequenced-verified short hairpin RNA (shRNA) for Aurora-A (TRCN000040044, Sigma-Aldrich) or an equivalent scramble sequence, accordingly with manufacturer’s instructions.

Lentiviral supernatants were collected 48 h following transfection of the packaging HEK-293T cells using X-tremeGENE DNA Transfection Reagent (Roche Applied Science). After addition of 8 μg/mL polybrene (Sigma-Aldrich) 3 × 10^5^ cells were infected, after 24 hours medium was replaced. For MCF-7 cells infection efficiency was evaluated counting GFP-positive cells. MDA-MB-231 or KBr2 positive cells were selected with 0, 5 mg/ml puromicin for 2 weeks (Sigma-Aldrich). AurkA overexpression or knock-down were confirmed by Q-PCR.

### Flow Cytometry

Cytofluorimetric analyses to evaluate CD44^+^/CD24^−/Low^ cells were performed as described in ref. 38. AldeFluor analyses was carried out accordingly to manifacturer’s protocol.

Results were processed with CellQuest Software (Becton-Dickinson, San Jose, CA) for statistical analyses. Statistical evaluation of positive cells was obtained by three independent experiments. Standard deviations is indicated.

### Protein Co-Immunoprecipitation

250 μg of lysate from MCF-7 or MDA-B-231 cells was used to perform a single Co-IP in 1 mL final volume using 30 μL of Sepharose G protein resin (GE-Healthcare). Rabbit anti-AurkA antibody (sc-25425, H-130; Santa Cruz Biotech) and pre-immune serum were previously immobilized on the resin. Resins were washed with wash buffer (50 mM Tris-HCl pH7) and incubated with 1 mg/mL BSA for 1 h at 4 °C. Resins and lysates were incubated for 3 h at 4 °C. Unbound proteins were removed with wash buffer.

Finally, proteins were eluted by SDS sample buffer (40 μL). 20 μL of the eluted proteins were loaded in a gel for SDS-PAGE to assess the presence of Snail after incubation with a Rabbit anti Snail (1:100, PA5-11923 Pierce).

## Additional Information

**How to cite this article**: Eterno, V. *et al*. AurkA controls self-renewal of breast cancer-initiating cells promoting wnt3a stabilization through suppression of miR-128. *Sci. Rep.* **6**, 28436; doi: 10.1038/srep28436 (2016).

## Figures and Tables

**Table 1:** Correlation between AurkA overexpression and clinical/pathological features of breast cancer tissues (N = 89).

		AurkA+ BCs
BCs, N = 89		46,07% (41/89)
Clinical Histotypes	Triple Negative	31,58% of TN *29,27%* (*12/41*)
	Luminal A–B	54,17% of Luminal *31,71%* (*13/41*)
	Her2	59,26% of Her2+ *39,02%* (*16/41*)
Grading	G1–G2	26,83% (11/41)
	G3	73,17% (30/41)
Ki67	>15%	63,42% (26/41)
	≤15%	36,59% (15/41)
Tumor Size (mm)^1^	<1	4,88% (2/41)
	1–2	36,59% (15/41)
	>2	56,10% (23/41)
	^1^ND	1/41
Linfonodal status (N)^2^	N0	39,02% (16/41)
	N1–3	56,10% (23/41)
	^2^ND	2/41
